# Avian Influenza H7N9 Virus Adaptation to Human Hosts

**DOI:** 10.3390/v13050871

**Published:** 2021-05-10

**Authors:** Swan Tan, Muhammad Farhan Sjaugi, Siew Chinn Fong, Li Chuin Chong, Hadia Syahirah Abd Raman, Nik Elena Nik Mohamed, Joseph Thomas August, Asif M. Khan

**Affiliations:** 1 Centre for Bioinformatics, School of Data Sciences, Perdana University, Wisma Chase Perdana, Changkat Semantan, Damansara Heights, Kuala Lumpur 50490, Malaysia; tanswan2011@gmail.com (S.T.); farhan@perdanauniversity.edu.my (M.F.S.); chong.lichuin@perdanauniversity.edu.my (L.C.C.); hadiasyahirah@gmail.com (H.S.A.R.); elena@perdanauniversity.edu.my (N.E.N.M.); 2 Department of Pharmacology and Molecular Sciences, The Johns Hopkins University School of Medicine, 725 North Wolfe Street, Baltimore, MD 21205, USA; siewchinn@hotmail.com (S.C.F.); jthomasaugust@gmail.com (J.T.A.); 3 Beykoz Institute of Life Sciences and Biotechnology, Bezmialem Vakif University, Beykoz 34820, Turkey

**Keywords:** influenza virus, zoonosis, H7N9, avian viruses, human viruses, host, adaptation, surveillance, diversity, motifs

## Abstract

Avian influenza virus A (H7N9), after circulating in avian hosts for decades, was identified as a human pathogen in 2013. Herein, amino acid substitutions possibly essential for human adaptation were identified by comparing the 4706 aligned overlapping nonamer position sequences (1–9, 2–10, etc.) of the reported 2014 and 2017 avian and human H7N9 datasets. The initial set of virus sequences (as of year 2014) exhibited a total of 109 avian-to-human (A2H) signature amino acid substitutions. Each represented the most prevalent substitution at a given avian virus nonamer position that was selectively adapted as the corresponding index (most prevalent sequence) of the human viruses. The majority of these avian substitutions were long-standing in the evolution of H7N9, and only 17 were first detected in 2013 as possibly essential for the initial human adaptation. Strikingly, continued evolution of the avian H7N9 virus has resulted in avian and human protein sequences that are almost identical. This rapid and continued adaptation of the avian H7N9 virus to the human host, with near identity of the avian and human viruses, is associated with increased human infection and a predicted greater risk of human-to-human transmission.

## 1. Introduction

Influenza A viruses belong to the *Orthomyxoviridae* family and circulate among aquatic wildfowl, which is their natural reservoir [1,2]. They mutate very rapidly as quasispecies [3], with over 100 subtypes based on different combinations of the external proteins, hemagglutinin (HA) and neuraminidase (NA) [4]. With many additional mutations of the other virus proteins that are selected by fitness in a given host [5], influenza viruses exist as a vast number of different strains that infect multiple bird and several mammalian species, including *Homo sapiens*. During the past century, more than ten of the avian virus subtypes were also infectious in humans [6]. Three (H1N1, H2N2, and H3N2) were capable of human-to-human (H2H) transmission and the cause of world-wide pandemics [4,7]. The initial H1N1 Spanish flu of 1918/1919 claimed over 40 million lives [2,7,8,9]. Other subtypes, such as H5N6, H6N1, H7N2, H7N3, H7N7, H9N2, H10N7 and H10N8, are capable of causing human infection as well [10]. The most recent human adapted subtypes, H5N1 and now H7N9, despite the lack of H2H spread, have also infected hundreds of people [11]. Fortunately, the relatively few human epidemics by highly infectious influenza A subtypes indicate that the vast majority of avian strains lack the critical sequences required for human infection and replication. Nevertheless, it is evident that without a new form of intervention, pandemics of human influenza infection, although rare, will continue. Despite the exigency of this risk, it is currently impossible to reliably predict the emergence of a new pandemic, and additional tools are needed for scientists and policymakers to evaluate the risk posed by influenza viruses.

In 2013, after decades of global distribution in avian hosts, specific strains of the H7N9 subtype influenza A virus were found to infect humans and have since infected over 1500 people with a mortality of approximately 40% [12,13,14,15,16,17,18,19,20,21,22,23,24]. The evolutionary history of the avian H7N9 virus is complex, involving point mutations and gene re-assortment with H7 and N9 viruses, and implicating multiple host species. The precise genesis and source of H7N9 viruses, thus far, remains unknown [25]. The internal genes are thought to be derived from avian H9N2 viruses, while the HA and NA genes are from unknown avian H7N?/H?N9 viruses of Eurasian origin [13]. The structural genes (HA and NA) later likely arose from avian H7N3 and H2N9/H11N9 viruses of Asian origin. There is even a suggestion that the polymerase basic 1 (PB1) segment of H9N2 may have recombined with the highly pathogenic H5N1 virus [26]. Domestic ducks are indicated to act as key intermediate hosts, serving as a reservoir of diverse influenza viruses, facilitating the generation of different subtype viruses, and transmitting them to chickens. The adaptation of the virus to humans in 2013 indicated a critical role of the recent genetic changes. The continued evolution of both avian and human H7N9 viruses has produced multiple strains more efficient as human pathogens.

Many studies of the genetic changes required for transmission of avian influenza viruses to human hosts (A2H) have been reported [23]. These include mutations of the basic polymerase 2 (PB2) protein that enhance viral RNA replication in human cells [27,28,29], and of haemagglutinin (HA) that facilitate avian virus binding to human cells [30,31]. There also has been a large-scale analysis of mutations associated with human-to-human (H2H) transmission of influenza A viruses [32,33]. However, a comprehensive spatio-temporal analysis of the incidence of the A2H H7N9 substitutions, and their transmission pathway between the avian and human virus populations, is lacking. Such data are needed for further elucidation of the biological mechanisms of viral adaptations to humans.

The focus of this work was to study all the changes or substitutions in protein sequences, irrespective of the evolutionary forces, such as mutation, genetic re-assortment and/or recombination, that resulted the changes. Thus, worldwide, recorded influenza A H7N9 avian and human protein sequences deposited at publicly available databases were retrieved at two time points (2014 and 2017) and analyzed. Herein, we describe a large-scale, quantitative analyses that compared the 4706 overlapping aligned nonamer amino acid sequences (1–9, 2–10, etc.) of the reported avian and human H7N9 strains present in datasets of 2014 and 2017. The analyses identified amino acid substitutions of nonamer sequences that distinguished the H7N9 strains recovered from avian and the newly reported human hosts in 2014, and it assessed the continued evolution of the avian and human viruses, as reported in 2017.

## 2. Results

### 2.1. Datasets of H7N9 Influenza Virus Sequences and Scope of the Analysis

The influenza H7N9 virus datasets of this study were obtained from the publicly available influenza specialist databases. The initial dataset collected in 2014 included 1031 H7N9 sequences from 18 avian hosts and 479 from humans. Those collected from avian hosts before the human infection in 2013 (599) were chiefly from domestic (indicated as only “duck” in the nomenclature) and wild ducks. The 432 sequences collected after 2013 were primarily from chicken hosts and included pigeon and tree sparrow hosts (Supplementary Table S1A). Unfortunately, no avian H7N9 virus sequences of chicken, pigeon (including wild and homing pigeon), or tree sparrow hosts were reported prior to human H7N9 infection in 2013. All human sequences (479) came from 15 cities located in China, Hong Kong, or Taiwan (Supplementary Table S1B). The 1031 avian virus sequences ranged from 77 PB1-F2 to 101 HA, and those of the 479 human virus sequences ranged from 30 PB1-F2 to 56 NA (Table 1). This range is because the reported viral data is a mixture of incomplete (a large majority) and complete genome strains.

The evolution of the human H7N9 virus of the 2014 dataset was further analyzed by comparison with a later (February 2017) and much larger dataset: 6436 avian and 8961 human H7N9 sequences (Table 2).

### 2.2. Protein Sequence Diversity of the 2014 Avian and Human H7N9 Virus Dataset

**Figure 1:**
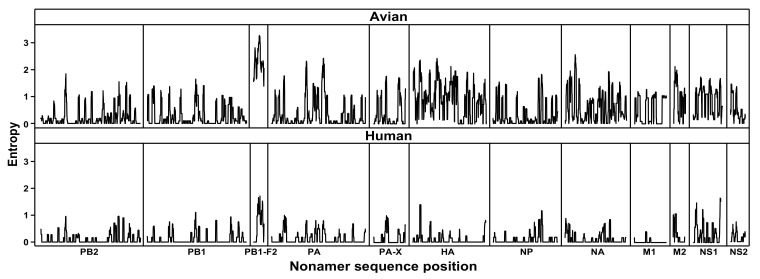
Protein sequence diversity of avian and human influenza A (H7N9) viruses. Shannon’s entropy was used as a general measure of protein sequence diversity for each aligned nonamer (nine amino acids) position of the H7N9 avian (upper) and human (lower) virus proteomes. The entropy values indicate the level of variability at the corresponding nonamer positions, with a zero representing completely conserved sites and high entropy values of about 3 or higher marking highly variable sites.

Shannon entropy [33,34,35,36] was used as a generic measure of protein sequence diversity for each aligned overlapping nonamer position of the avian and human H7N9 viral proteomes (Figure 1). Entropy of a given position represented the number and individual incidence of the different nonamer sequences at the position. The avian H7N9 virus proteins, with an evolutionary history of over 25 years, were markedly diverse. PB1-F2, with substitutions at each of the aligned nonamer positions, was highly diverse, and NS1, NS2, and M2 each had less than 10 completely conserved positions. The more recent human H7N9 viruses (post-2012), in contrast, had relatively few substitutions and contained numerous long stretch of regions of nonamer positions with no substitutions (zero entropy). Nevertheless, despite the limited history, all proteins of the human H7N9 viruses contained regions of nonamer sequence diversity.

### 2.3. Quantitative Analysis of Avian and Human H7N9 Virus Protein Sequence Diversity of the 2014 Dataset

The 1031 avian and 479 human H7N9 protein sequences of the 2014 dataset were aligned, and each of the 4706 overlapping nonamer positions of the avian and human virus proteomes were analyzed for both amino acid substitutions and the avian hosts of viruses containing these substitutions (Supplementary Table S2).

Each nonamer sequence present in the 4706 overlapping nonamer positions of the aligned avian and human H7N9 virus proteomes was classified as a defined diversity motif based on the incidence of the sequence in the aligned viruses: (a) the most prevalent or “index” sequence; (b) the second most prevalent and dominant substitution of the index sequence as the “major variant” sequence; (c) “minor variants”, other index sequence substitutions, each with an incidence less than that of the major variant, and observed more than once in the aligned viruses; and (d) “unique variants”, substitutions observed only once in the aligned virus sequences. Supplementary Table S2 provides data describing all of the distinct sequences at each of the 4706 overlapping nonamer positions, including their diversity motif assignments, and the avian host species harboring the viruses with the distinct nonamer sequences.

Overall, the avian and human H7N9 virus proteins of the 2014 datasets had identical index sequences at ~83% (3923) of the 4706 proteome nonamer positions. An example of positions with the same index sequence in both avian and human H7N9 viruses is the HA protein alignment position 1–9 (Table 3). MNTQILVFA, the index sequence, was present in ~75% of the avian viruses. Substitutions (~25% total) of the avian index sequences were present in several forms, primarily as the “major” variant (~16%) chiefly found in turkey viruses, “minor” variants (~6% total) present in turkey viruses and those of other hosts with less than 2% individual incidence, and two “unique” variants (~1% each) present in duck (domestic, if not indicated) and another host. The index sequence (MNTQILVFA) of the 40 human H7N9 viruses at this position was the same as that of the avian viruses and without substitutions (100% incidence; completely conserved).

The remaining ~17% of the 4706 proteome nonamer positions had index sequences that differed between the avian and human viruses by one or more amino acids. An example is the HA nonamer position 227-235 that contained the previously reported HA Q235L (glutamine to leucine) avian-to-human H7N9 substitution [13] (Table 3). The avian index sequence (GARPQVNGQ) was present in ~63% of the avian and none of the human H7N9 viruses. Rather, the Q235L substitution of the avian viruses, a major variant present in ~35% of the 99 aligned avian H7N9 viruses was selectively adapted as the corresponding human virus index sequence with ~93% incidence in the reported population. The remaining ~7% of human viruses contained three unique variant substitutions not observed in avian viruses (human-specific substitutions).

Metadata of this analysis included the host species of the avian virus strains and geographical distribution for the human H7N9 viruses (Supplementary Tables S1A,B and S2). The chicken was the predominant host of avian H7N9 viruses with shared identity to index sequences of human viruses. In contrast, the hosts of the avian H7N9 viruses with index sequences that were different from the human viruses were mainly of duck (domestic), wild duck, turkey, and several others with low H7N9 virus incidences (Supplementary Table S2).

### 2.4. Avian H7N9 Major Variant Substitutions as Human H7N9 Virus Index Sequences

**Figure 2:**
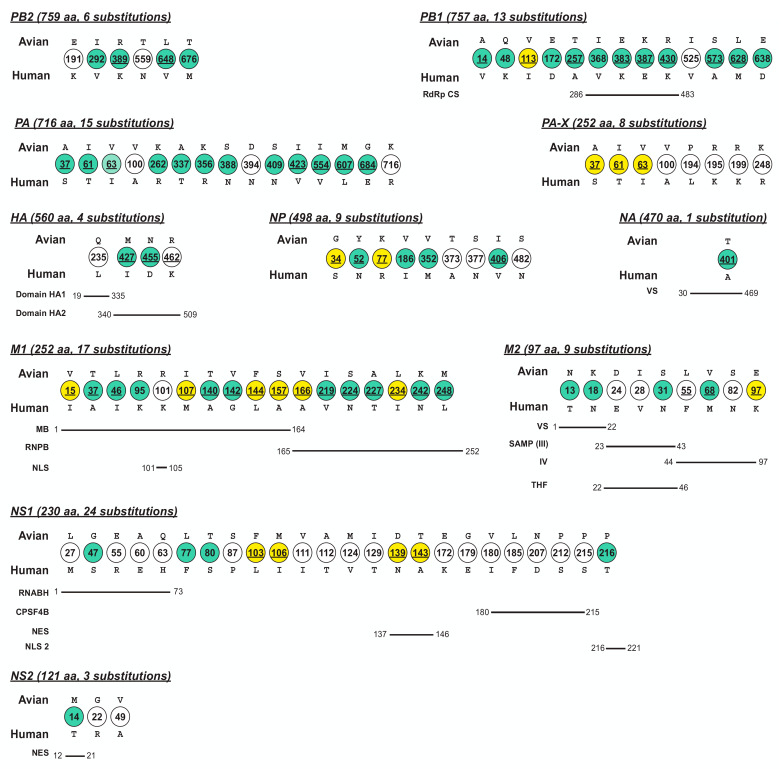
Avian-to-human (A2H) substitution identified in the proteins of influenza A (H7N9) viruses. The amino acid positions of the A2H substitutions are indicated in the circles, and those underlined are the 50 that remained unchanged in the recorded human H7N9 population. The circles in green shade are substitutions that occurred in the evolutionary path of A (H7N9) viruses [16]; while those in yellow were first detected in 2013. The protein numeration is based on protein sequence alignment. Abbreviations: RdRp CS, RdRp catalytic subunit; HA, hemagglutinin; VS, virion surface; MB, membrane binding; RNPB, ribonucleoprotein binding; NLS, nuclear localization signal; SAMP (III), signal-anchor for type III membrane protein; IV, intravirion; THF, transmembrane helical fragments; RNABH, RNA-binding and homodimerization; CPSF4B, cleavage and polyadenylation specificity factor 4 binding; and NES, nuclear export signal.

A key observation from the 2014 dataset was the presence of 109 major variant substitutions of avian H7N9 proteins that distinguished the avian and human strains. These substitutions, with incidences, by definition, not exceeding 50% in the avian viruses, were selectively adapted as the corresponding human H7N9 index sequences, with initial incidences of 100%, before any substitutions (Figure 2, Supplementary Table S3A–D). These A2H substitutions were distributed among each of the virus proteins except PB1-F2. They occurred primarily in NS1 and M1, and to some extent M2 and NS2, with an average of one substitution every 8 to 18 amino acids. Some appeared clustered with as many as three substitutions in a single nonamer position. Many of the substitutions overlapped reported functional sites of the proteins. In contrast, the substitutions were less frequent, every 30–56 amino acids, in PA, PA-X, NP, and PB1, and least in HA and PB2, every 75–97 amino acids.

About one-half (53) of the 109 A2H substitutions were long-standing in the historical evolution of the avian H7N9 as previously reported [16] (Figure 2, green highlight). Notably, 17 of the remaining A2H substitutions (Figure 2, yellow highlight) were first reported in 2013. These 17 substitutions were distributed in six of the H7N9 proteins, with the largest concentrations in M1 (6) and NS1 (4) (Figure 2), and were possibly required for human infection.

With the continued, rapid evolution of the human H7N9 viruses, only 50 of the 109 A2H substitutions remained without change (completely conserved; 100% incidence) in the 2014 human dataset (Figure 2). These 50 substitutions were distributed with greatest representation in M1 (15 of the 50 A2H) and to a lesser extent in the remaining proteins, except for NS2 and PB1-F2 (Supplementary Table S3A–D). The remaining 59 of the 109 A2H substitutions were present in ~66–98% of the human viruses, having been replaced by changes (~2–34%) (Supplementary Table S2).

### 2.5. Avian Host Source of Human H7N9 Influenza Viruses

**Figure 3:**
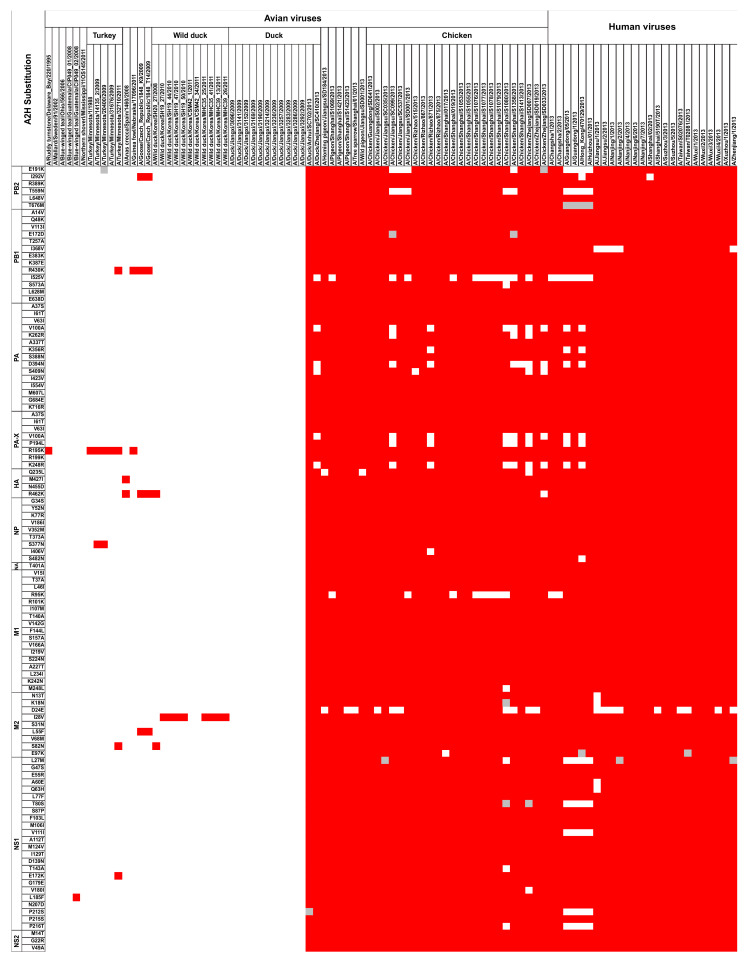
Heat map depicting the distribution of the 109 identified avian-to-human (A2H) substitution sites (rows) of publicly reported, full-length, avian and human influenza A (H7N9) virus strains (columns). The identified A2H amino acid (a.a.) substitutions are sorted according to the influenza A virus segments. The distribution is shown with red representing the presence of the A2H a.a. substitution (human index), white for avian index, and grey for strains that exhibited neither (i.e., other variants) or the presence of a gap at the respective position. Eurasian teal is referred to here with the scientific name *Anas crecca*. Do note that for the strain A/Goose/Czech Republic/1848_K9/2009, the complete proteome sequence was taken from FluDB, while for the other strains, the PA-X sequence was from FluDB and the other proteins were from GISAID. Full-length strains that could not be ascertained by the accession were ignored.

All of the 109 A2H substitutions of the 2014 dataset, present as major variant sequences of avian viruses, were found in a large fraction (~19–35%) of avian viruses of the chicken host (Supplementary Table S3A–D). These substitutions were also present in a small fraction (~1–4% each) of several (12) other avian H7N9 hosts (Figure 3), primarily domestic duck, pigeon (including wild and homing pigeon), and tree sparrow. Moreover, besides chicken, only H7N9 viruses of domestic duck (collectively as a group) and pigeon (collectively) contained each of the 109 substitutions (Figure 3). Viruses of wild pigeon, tree sparrow, and homing pigeon hosts were missing one, one, and two substitutions, respectively. The high incidences of the 109 A2H substitutions in the H7N9 viruses of chicken suggest a selective advantage of the substitutions in this host, as well as in humans.

The distribution of the 109 A2H substitutions of the 2014 dataset, analyzed in individual, full-length genome of avian (69) and human (25) viral strains, showed that all or a majority of the substitutions were found almost exclusively in individual viruses of human (25/25) and chicken (24/24) hosts, and were also observed notably in a few of the other avian hosts, namely domestic duck (2/13), pigeon (3/3), wild pigeon (1/1), homing pigeon (1/1), and tree sparrow (1/1) (Figure 3). Prominently, all avian and human viruses that contained the majority or the complete set of the 109 substitutions were reported in the year 2013. Alas, there were no chicken H7N9 viruses reported before 2013. However, the domestic duck, which had 11 full-length, H7N9 genome sequences reported before 2013, had none that exhibited any of the A2H substitution. It is apparent that the genetic change(s) that occurred in 2013 affected multiple avian hosts, in addition to the chicken. Prior to 2013, specifically from as early as 1988 to 2011, H7N9 viruses of a few avian hosts exhibited limited A2H substitutions, one to four of 11 in each virus (PB2: I292V; PB1: R430K; PA-X: R195K; HA: M427I and R462K; NP: S377N; M2: I28V, L55F, and S82N; NS1: E172K and L185F) (Figure 3). These hosts were namely ruddy turnstone (one A2H substitution in one viral sequence from Delaware Bay, USA isolated in 1995), blue-winged teal (one A2H substitution in one viral sequence from Guatemala, isolated in 2008), turkey (five A2Hs, distributed over five viral sequences from Minnesota, USA, isolated in 1988, 2009, or 2011), and Eurasian teal (referred to with the scientific name *Anas crecca* in some figures or supplementary files) (two A2Hs in one viral sequence from Spain of 2008), guinea fowl (two A2Hs in one viral sequence from Nebraska, USA of 2011), goose (four A2Hs, each in two viral sequences from the Czech Republic, isolated in 2009), and wild duck (three A2Hs, distributed over nine viral sequences from Korea, isolated in 2008, 2010, or 2011). Taken together, these data suggest that the substitutions of chicken H7N9 viruses that occurred in 2013 enhanced the distribution of the virus in multiple other avian hosts, as well as humans.

The absence of many of the substitutions in several of the human virus strains of the 2014 dataset suggested that not all 109 A2H are required for infection and/or replication in human hosts. For example, the human strain A/Guangdong/05/2013 possessed only 94 of the 109 A2H substitutions. This suggests that only the 50 A2H substitutions that were present in all human viruses may be critical for survival in the human host (Supplementary Table S3A–D). Notably, 19 of the 50 A2H substitutions with an incidence of 100% in the human viruses did not correspond to ancestral changes of H7N9, and all but two (M2: L55F and HA: R462K) were first reported in 2013 (Supplementary Table S4). These 17 are, thus, candidates for substitutions in avian viruses that were essential for H7N9 adaptation to human hosts.

### 2.6. Substitutions Specific to the Human H7N9 Viruses (H2H)

**Figure 4:**
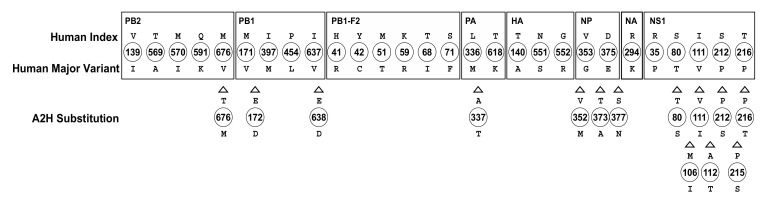
Major variant amino acid substitutions specific to human influenza A (H7N9) virus (H2H). The amino acid positions of the substitutions are indicated in the circles. Each site represents a major variant substitution, with an incidence of 10% or more, to the human virus index sequence. Some of the substitution sites are at close proximity (8 amino acids) or share the same amino acid position as the indicated avian-to-human (A2H) substitutions. Refer to Supplementary Table S3A–D for all A2H sites and Supplementary Table S5 for all H2H sites.

Despite the limited evolutionary history of the human H7N9 viruses, substitutions specific to the human viruses (H2H; 188; 2014 dataset) were distributed among each of the virus proteins (Supplementary Table S5). The majority were unique variants, each present in only one (~2–4%) of the aligned human viruses, and thus not selective in the virus population. However, 28 specific substitutions of the human H7N9 viruses were major variants with ~10% or more incidence (Figure 4) and found in viruses from multiple geographical regions of China, Hong Kong, and Taiwan, indicating a selective fitness of the substitutions in human hosts. In addition, 50 of the 188 human-specific substitutions, particularly those of NS1, PA, M2, and PB1, occurred at the same, or adjacent to, amino acid positions of the A2H substitutions, suggesting additional fitness adaptation of protein sequences involved in avian-to-human transmission (Figure 4 and Supplementary Table S5).

### 2.7. Continued Evolution of the Human H7N9 Viruses (2017 Dataset)

**Figure 5:**
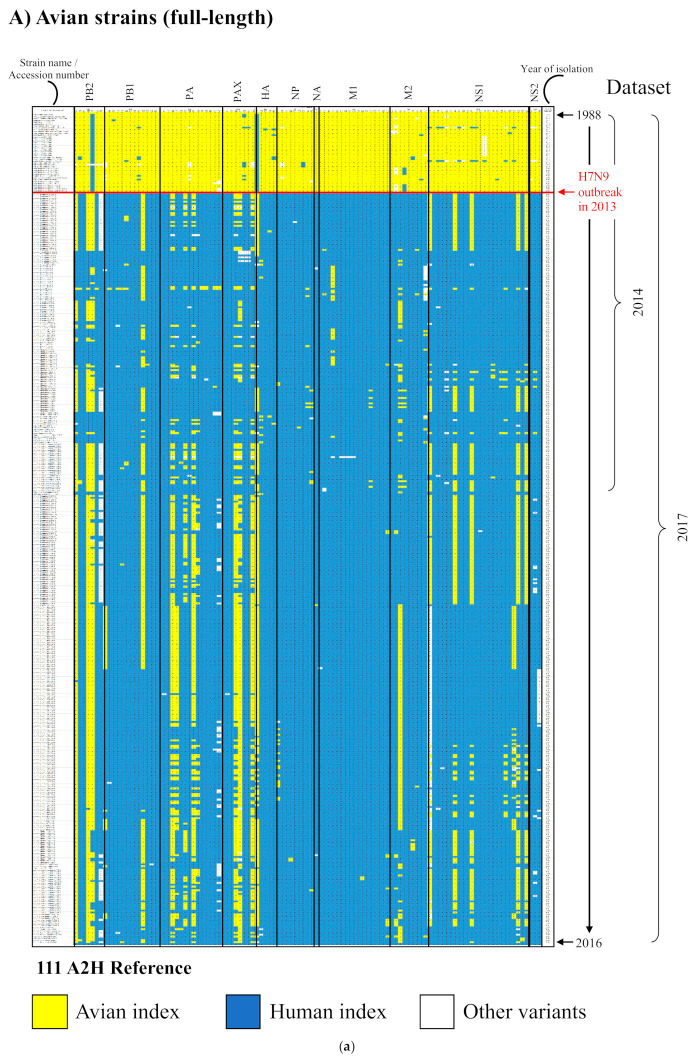

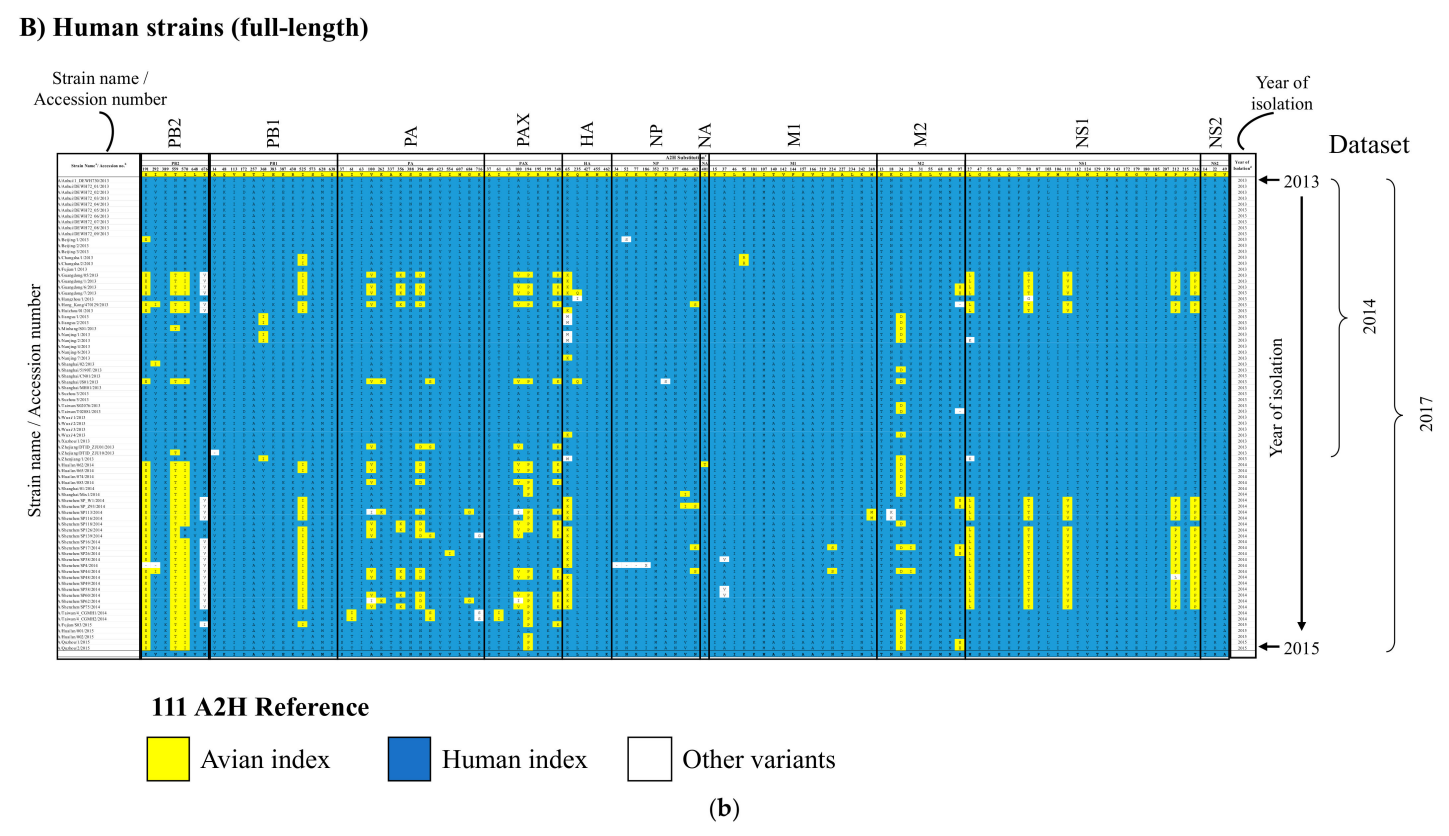
Heat map depicting timeline of adaptation to avian-to-human substitution (A2H) for all proteins of influenza A among publicly reported, full-length avian and human influenza A(H7N9) virus strains. Using the 109 A2H substitution sites identified from the 2014 dataset and the two new sites from 2017 dataset as a reference, corresponding signature residues for each reported avian (**A**) and human (**B**) H7N9 strain sequence are shown in alignment to the reference and arranged in chronological order of strain isolation (up to 2016 for avian strains and 2015 for human strains; only strains reported with full-length protein sequences were analyzed; full-length strains that could not be ascertained by the accession were ignored). The signature columns within each protein show the residue observed at each of the A2H substitution sites. Each strain is annotated with subtype, year and country of isolation, and isolate name. The first and the last pattern of the alignment are the avian-to-human substitution (A2H) residues, with the avian index sequence as the first (top) pattern and the human index sequence as the last pattern (bottom). Signature residues characteristic of the avian index are shown on a yellow background, while residues characteristic of the human index are shown on a dark blue background, and all other variants are on white. A higher resolution of the image, with visible details, is provided in Supplementary Table S7.

The identified 109 A2H amino acid substitutions were assessed for their evolutionary stability in the larger dataset of 2017. Only seven remained as avian, major variant amino acid substitutions selected in the human host, as index sequences (Supplementary Tables S3A–D and S6). These seven were present in four proteins: PB1 (I525V), PA (V100A, D394N), PA-X (V100A, P194L, K248R), and NS1 (L27M). The avian host distribution of these seven A2H substitutions in the 2017 dataset remained largely the same as the 2014 dataset, present in chicken (chiefly, with increasing incidence in nearly all cases), domestic duck (incidence largely maintained), pigeon (including wild and homing pigeon; incidence decreased), tree sparrow (incidence decreased), with expansion to one additional host, goose (I525V). Additionally, there were two new A2H substitutions, I570M and K65R, observed in PB2 and HA, respectively (Supplementary Tables S3A–D and S6). Of the remaining 102 (out of the 109) substitutions “lost”, 95, which previously (2014 dataset) were avian, major variant amino acid substitutions, became established as the avian index (2017 dataset) (Supplementary Table S6), while the remaining seven (of 102) were no longer the index sequences among the human viruses. The 109 early and two new substitutions were visually depicted by use of a heat map for all proteins of influenza A among publicly reported, full-length avian and human influenza A(H7N9) virus strains (Figure 5; Supplementary Table S7 for high resolution) to illustrate the timelines of adaptation to avian-to-human substitution. The illustration revealed that all of the avian strains (since 2013 outbreak), individually, had a majority of the positions as characteristic of the human index (shown on a dark blue background). Additional analysis of the data using Euclidean clustering showed a clear separation between the avian strains, before and after the 2013 H7N9 outbreak, with two major subclusters among the 2013 onward viral strains (Supplementary Figure S1).

### 2.8. A2H Substitutions between H7N9 and H9N2

The A2H of the internal proteins of H7N9 were compared for correspondence to the internal proteins of H9N2 chicken viruses. Majority (~88%) of the H9N2 sequences before 2013 exhibited the A2H substitutions (Supplementary Table S8). This trend (~91%) continued with the 2013 onward sequences.

## 3. Discussion

The complexity of protein substitutions associated with avian H7N9 virus infection of humans is revealed by this *in silico* finding of 109 A2H substitutions that were selectively present in the initial human H7N9 viruses. The A2H substitutions identified may be as a result of mutation, re-assortment, or recombination, which merit further investigation. Each was the most prevalent, major variant substitution at a given nonamer position of the aligned avian H7N9 viruses that was adapted as the most prevalent index sequence at the corresponding nonamer position of the aligned human H7N9 viruses, with an incidence of 100% before the onset of change. About one-half of the 109 A2H substitutions were long-standing in the historical evolution of H7N9, as previously reported in phylogenetic studies [16]. Thus, although possibly required, they were not sufficient for human infection and can be considered to be adventitious selections with respect to the human host. Moreover, many (59) of the original 109 substitutions were replaced to some extent by sequence changes in the H7N9 viruses recovered from infected humans. For example, the substitution Q235L, known to be selective in human viruses for receptor specificity to human α2,6 sialic acid [37], was replaced by unique variants in two human strains.

Three of the A2H substitutions (HA: Q235L (H7 numbering); PB1: I368V; and M2: S31N) were reported by Gao et al. to be present in influenza A (H7N9) viruses associated with the first three human infections (A/Shanghai/1/2013; A/Shanghai/2/2013; and A/Anhui/1/2013) in China, early 2013, which were fatal. Nine matched to 68 human adaptation signature sites identified from several subtypes (H1N1, H2N2, H3N2, H5N1, H9N2, H6N1) by Miotto et al. 2010 [33]. Additionally, experimental findings of the CDC weekly report [38] noted the two HA amino acid residues, 186V and 226L/I in H3 numbering (177 and 217 in H7 numbering) and PB1-368V, are likely to increase human receptor binding and enhance transmission to humans [39], respectively.

Possibly, only the 50 A2H substitutions present in all human H7N9 viruses in the 2014 dataset may be essential for human adaptation. Notably, 17 of these 50 were first recorded in 2013. These 17 substitutions were particularly abundant in two proteins: the M1 matrix protein that mediates nuclear export of viral RNA segments [40] and is thought to initiate progeny virus assembly and budding [41], and NS1 that is associated with an increased translational rate of viral mRNAs [42] and suppression of the host immune response [43,44]. The data suggest that screening of animal influenza A viruses for threat of crossing to humans should not be limited to only the surface proteins.

Multiple avian species were the host origin of the 109 A2H substitutions associated with the 2013 human-adapted H7N9 viruses. While the chicken contained the largest fraction of avian viruses with the 109 A2H substitutions, five other hosts (domestic duck, pigeon, wild pigeon, homing pigeon, and tree sparrow) contained a few reported H7N9 viruses with all or nearly all of the 109 H7N9 A2H substitutions. Remarkably, these hosts represent several unrelated avian families (*Anatidae*, *Columbidae*, and *Passeridae*), besides the chicken (*Phasianidae*). All H7N9 A2H substitutions from viruses of these five hosts were reported in 2013, and where data were available, the substitutions were not present in reported viruses of the same host prior to 2013, suggesting that adaptation to the chicken, pigeon, and tree sparrow accompanied the adaptation to humans. We hypothesize that the root cause for the genesis of the A2H substitutions in the chicken host in 2013 was also responsible for its distribution in other avian species. Unfortunately, information on the species evolution is limited for lack of data, particularly for the chicken, as no sequence data of H7N9 viruses from chickens were available prior to the year 2013.

The internal genes of H7N9 are thought to be derived from avian H9N2 viruses, while the HA and NA genes are from unknown avian H7N?/H?N9 viruses of Eurasian origin. Majority of the H9N2 sequences before 2013 exhibited A2H substitutions. This trend continued with the 2013 onward sequences. This supports the notion of H9N2 being the origin for the internal genes with the possibility of subsequent changes bringing about the additional substitution.

Prior to 2013, from as early as 1988 to 2011, H7N9 viruses of a few avian hosts (ruddy turnstone, blue-winged teal, turkey, Eurasian teal/*Anas crecca*, guinea fowl, goose, and wild duck) exhibited limited (11, collectively, Figure 3) A2H substitutions. In the genesis of H7N9, domestic ducks have been proposed to act as key intermediate hosts, facilitating the generation of different subtype viruses, and transmitting them to chickens [13]. H7N9 viral sequence data from domestic duck prior to 2013 were only available for the years 2008 (three HA and one NA; all from Mongolia), 2009 (11 full-length viral genome sequences, all from Jiangxi, China), 2010 (one HA, Mongolia), and 2011 (one HA, Gunma), all of which did not exhibit any of the A2H sites. The A2H sites were only mapped in the available viral genomes of domestic ducks (two isolates, Anhui and Zhejiang, China) starting in 2013, which is the same year they were observed in chickens. Although seven (collectively) of the A2H substitutions were missing from one of the two domestic duck viral genomes of 2013 (Figure 3), all the seven, except two (S409N in PA and P212S in NS1; Figure 2), were also missing in more than one strain of chicken viruses, as well as human viruses. The two A2H substitutions were either missing in chicken or human viruses.

The substitution T401A in the second sialic acid-binding site of neuraminidase (NA) protein, which is an important factor in the hemagglutinin–neuraminidase receptor balance [45], is indicated to enhance catalytic activity, functionally mimicking the substitutions of avian-derived influenza A viruses that became pandemic in humans [46]. This substitution was observed in all the full-length strains of human, chicken, wild pigeon, tree sparrow, pigeon, homing pigeon, and domestic duck (Figure 3). Phylogenetic analyses revealed that the substitution T401A occurred prior to those in hemagglutinin (HA), suggesting that the substitution may have facilitated the acquisition of altered HA receptor-binding properties and contributed to the spread of the novel H7N9 viruses, which still continue to pose a public health threat.

We speculate that H7N9 chicken viruses prior to 2013 did harbor a number of the 109 A2H substitutions, given that at least 12 other hosts did exhibit a few. The 109 A2H substitutions, however, were completely absent from reported 2008-2011 H7N9 viruses of domestic ducks, a species proposed as a key intermediate host in transmitting to chickens [13]. Given that 2013 H7N9 viruses of domestic ducks closely mirrored the distribution of A2H substitution in chicken viruses of the same year, it is likely that 2008–2011 H7N9 chicken viruses also closely mirrored the absence of A2H substitutions. It is quite possible that domestic ducks and chickens started exhibiting the A2H substitutions from 2011 onward, leading up to the emergence of the 2013 H7N9 strain. This may have particularly involved about one-half of the 109 A2H substitutions that were long-standing in the historical evolution of H7N9; only 17 of the A2H substitutions were first reported in 2013. Nevertheless, the available data indicate that several avian hosts now possess greater potential for human H7N9 infection if additional substitution(s) enhance the fitness and frequency of the A2H substitutions. These findings call for wider surveillance of the avian host species, particularly domestic ducks given their extensive farming.

The widely reported PB2 E627K substitution [27,28,29] of H7N9 and other human influenza viruses, important for enhancement of replication, is not reported herein as an A2H substitution because it did not conform to the common pattern of an avian major variant selectively adapted as the corresponding human index substitution. The E627K substitution is found in avian species only as a unique variant (incidence ~1%) of the tree sparrow, whereas it is the dominant sequence in human hosts (incidence ~68%), likely as a result of subsequent sequence changes of the infecting virus in humans rather than the avian host [29].

Despite the short evolutionary history of the human H7N9 viruses, there is rapid and continued fitness evolution of the virus in human hosts. In this study, over 200 human H7N9-specific substitutions, not present in the avian H7N9 viruses, were identified. Several were adjacent to or overlapping the positions of the A2H substitutions. In the absence of human-to-human transmission, there is little selective pressure for the proliferation of the human virus strains.

The evolution of the 109 substitutions was analyzed by comparing the 2014 datasets (avian and human) with the much larger 2017 datasets (avian and human). Only seven of the original A2H substitutions remained in the 2017 sequences, with two that were newly identified. The absence of the 102 substitutions does not represent that they are lost, but rather, that the originally selected major variant substitutions of the avian viruses have further adapted in avian hosts and have become widespread in the population as the index of the avian H7N9 sequences. Thus, in the recent 2017 dataset, many of the 2014 major substitutions had become the index in both avian and human viral strains, and hence the lack of apparent selection between the two viral populations. This observation was not restricted to viral strains of chicken, which were predominantly sequenced, and thus a potential bias, but extended to other hosts. The sub-clustering among the 2013 onward strains indicates further evolution and possible adaptation into multiple lineages. These results highlight the need for stratification of viral sequence data in a time-series fashion as a better strategy for identification of A2H substitutions and understanding the transmission patterns.

In summary, the data indicate a remarkably rapid and continued A2H fitness evolution of the avian H7N9 viruses in avian hosts (chicken, domestic duck, pigeon, wild pigeon, homing pigeon, and sparrow), in particular the chicken. This correlates with the progressive increase in the number of people infected by the virus since 2013 [24], with annual epidemics of human infections increasingly reported in China, where it experienced its fifth (October 2016 to September 2017) and largest epidemic (766 infections) [24,47], which was followed by the sixth epidemic [24]. As essentially all chickens in China are now possibly hosts of the human H7N9 strain, the exposure of humans to chickens should be limited, with continued surveillance, as necessary steps to monitor, curtail and/or prevent further spread and the possible emergence of new lineages capable of human-to-human transmission.

## 4. Material and Methods

### 4.1. Data Collection and Processing

All worldwide, recorded influenza A H7N9 avian and human protein sequences (from both complete and incomplete genomes) deposited at the publicly available database, Influenza Research Database (IRD; www.fludb.org; (accessed on 24 April 2014)), were retrieved for analysis by use of the Protein Sequence Search function. Protein sequence data (from both complete and incomplete genomes) for the avian and human H7N9 viruses were downloaded again in February 2017 for validation purposes, by pooling from two major flu specialist databases, the IRD and GISAID EpiFlu (http://platform.gisaid.org/epi3/; accessed on 12 February 2017) [48]. The data for 2017 were processed separately from the 2014 dataset, but using identical procedures. Similarly, influenza A H9N2 chicken protein sequences were retrieved from IRD as of October 2020 using the same procedures.

Data processing involved removal of redundant sequences for each protein, if the duplicates came from the same species. The remaining sequences of each protein from both avian and human were co-aligned by use of ClustalOmega [49] to allow for corresponding amino acid position comparison between the two groups. Partial (incomplete) sequences were included in the alignment because they provided additional data for the study of diversity. All multiple sequence alignments were manually inspected and corrected for misalignments. Alignment positions with high fractions, 95% or more, of gaps (insertions or deletions) were removed to minimize alignment errors. The protein alignments were then split to separate the human data from the avian; the co-aligned positions allowed comparative analysis.

### 4.2. Shannon’s Nonamer Entropy

Shannon’s entropy [34], applied to aligned overlapping peptides of size nine (1–9, 2–10, 3–11, etc.), was used as a general measure of avian and human H7N9 proteome sequence diversity, as described in Khan et al. 2008 [36] and others [50,51]. The sliding window approach of size nine was used for statistical significance and analysis of diversity in the context of the immune response (antigenic diversity) [52]. Each of the aligned overlapping nonamers represented a possible antigenic core binding domain for human leukocyte antigen (HLA; human MHC) molecules and T-cell receptors. This assumption is based on the fact that there is a large array of HLAs with different binding specificities in the human population [53]. Further, the repeated associations of each amino acid in a moving, overlapping 9-mer window can facilitate the detection of possible sequencing errors. Briefly, peptide entropy *H*(*x*) for each of the nonamer positions (*x*) in the protein alignments was computed by

(1) $$H\left(x\right)=\overset{n\left(x\right)}{\underset{i=1}{\sum }}p\left(i,x\right)log_{2}p\left(i,x\right)$$

where *p*(*i*,*x*) is the probability of a particular nonamer peptide *i* at position *x*, and *n*(*x*), the total number of peptides observed at the position. Although there are other methods to study sequence diversity, Shannon’s entropy applied to aligned nonamers was used because it readily provides the components (*p*(*i*,*x*) and *n*(*x*)) necessary for the subsequent proteome-wide quantification of the diversity motifs. Entropy values were corrected for data size bias by following the method described in Khan et al., 2008. Only sequences that contained a valid amino acid at position *x* were used for the entropy computation and subsequent analyses. Sequences that contained gaps (-) or any of the unresolved characters, including B (asparagine or aspartic acid), J (leucine or isoleucine), X (unspecified or unknown amino acid), and Z (glutamine or glutamic acid), were also excluded.

### 4.3. Quantitative Analyses of Diversity Motifs

The distinct sequences at each aligned nonamer position, for both the avian and human viruses, were classified as defined diversity motifs (index, major variant, minor variants, and unique variants) based on their incidence (% occurrence), as previously described [51]. The diversity motifs and their incidences at each of the nonamer positions allow evaluation of the substitution transmission dynamics and selectivity of the sequences in relation to the animal (i.e., avian) or human hosts of the virus. The in-house g-FLUA2H web-application was used to automate the motif assignment [52].

The aligned nonamer positions of the proteomes were subjected to a two-category chi-square test of goodness-of-fit, comparing the incidences of the avian H7N9 index and its major variant sequences against the incidences of the corresponding sequences of the human H7N9 viruses. The A2H substitution sites were identified by statistically significant differences of incidences of compared nonamer sequences at *p* < 0.05, with multiple test correction for alpha inflation by use of the Benjamini–Hochberg method [54]. Because a single amino acid substitution can affect nine overlapping nonamers spanning a region of 17 amino acids, and also given that motif switching of nonamer sequences (change of incidence across positions resulting in sequence rank change, and thus, motif change) has been reported for viral quasispecies populations [51], the sites were manually inspected for representative nonamer positions with avian major variant amino acid substitutions selected in the human host as the index sequence. These steps were also repeated on the February 2017 final processed data.

The nonamer sequences containing the selected A2H substitutions were annotated with known and putative structural and functional properties of the corresponding proteins by searching the literature and public databases Prosite [55], via ScanProsite [56], and Pfam [57].

### 4.4. Substitutions Specific to the Human H7N9 Viruses (H2H)

Substitutions specific to the human H7N9 viruses were identified by scanning for nonamer positions where a sequence, variant to the index was present in the human viruses, but absent in the avian viruses. Such nonamer positions that were overlapping and contiguous were manually inspected to select for a representative. Such substitutions that were unstable (change of incidence across positions resulting in sequence rank change, and thus, motif change) [51] and found within the first few amino acids of the N-terminal were ignored.

### 4.5. Comparison of the 109 A2H Substitutions between 2014 and 2017 H7N9 Datasets, and 2020 H9N2 Dataset

The identified 109 A2H amino acid substitutions from the 2014 dataset were assessed for their evolutionary stability in the larger dataset of 2017. This was performed by evaluating the net motif change in the substitutions between the two datasets, with three status forms: “Unchanged”, “New”, and “Reversed”.

All viral strains of both datasets that contained the full-length proteome sequence were extracted to construct a heat map that represented the timeline of the adaptation for all the 109 and the two new A2H amino acid substitutions. Further, an additional heat map with clustering was constructed by use of the R heatmap.2 function (gplots package) with the default Euclidean clustering (by row—strain name) option and a dendrogram tree. As a separate analysis, the A2H substitutions of the internal proteins were assessed for presence in H9N2 chicken viruses, before and 2013 onward, to evaluate the notion of H9N2 being the origin for the H7N9 internal genes.

## Figures and Tables

**Table 1:** Influenza A (H7N9) protein sequences analyzed from the 2014 dataset. The individual protein sequences of the 1031 avian viruses ranged from 77 PB1-F2 to 101 HA, and those of the 479 human virus sequences ranged from 30 PB1-F2 to 56 NA.

RNA Segment ^†^	Proteins *				No. of Sequences ^||^	
	Protein ^‡^	Abbreviation	Amino Acids	Nonamer Positions ^§^	Avian	Human
1	Polymerase basic 2	PB2	759	751	84	43
2	Polymerase basic 1	PB1	758	749	85	36
		PB1-F2	90	82	77	30
3	Polymerase acidic	PA	716	708	84	36
		PA-X	252	244	80	36
4	Hemagglutinin	HA	560	552	101	53
5	Nucleocapsid	NP	498	490	86	37
6	Neuraminidase	NA	470	462	92	56
7	Matrix 1	M1	252	244	86	38
	Matrix 2	M2	97	89	84	38
8	Non-structural 1	NS1	230	222	87	38
	Non-structural 2	NS2	121	113	85	38
**Total**				4706	1031	479

* Reference strain accession number: A/Anas crecca/Spain/1460/2008. The common name of the species is Eurasian teal. † The 8 segments of viral RNA encoding the corresponding proteins. ‡ Proteins encoded by the viral RNA segments. § The number of overlapping nonamer positions. || The number of avian and human protein sequences in the dataset retrieved as of 24 April 2014.

**Table 2:** Influenza A (H7N9) protein sequences from the 2017 dataset.

RNA Segment	Proteins		No. of Sequences ^||^	
	Protein	Abbreviation	Avian	Human
1	Polymerase basic 2	PB2	518	805
2	Polymerase basic 1	PB1	519	798
		PB1-F2	503	790
3	Polymerase acidic	PA	518	798
		PA-X	485	90
4	Hemagglutinin	HA	634	823
5	Nucleocapsid	NP	520	798
6	Neuraminidase	NA	623	823
7	Matrix 1	M1	523	809
	Matrix 2	M2	522	810
8	Non-structural 1	NS1	536	808
	Non-structural 2	NS2	535	809
**Total**			6436	8961

|| The number of avian and human protein sequences in the dataset retrieved as of February 2017.

**Table 3:** Samples of aligned HA nonamer position sequences of avian and human influenza A (H7N9) viruses. The nonamer position 1–9 is an example of a site where the index sequence is identical between the avian and human H7N9 viruses. In contrast, the nonamer position 227–235 is a sample site where the index sequence is different between the avian and human viruses, by one or more amino acids.

Position ^†^	Nonamer Sequence (Avian & Human) ^‡^	Avian Virus Sequences ^^^						Human Virus Sequences ^^^				
		Number of Sequences ^*^	Motif ^§^ (Incidence, %)	Avian Host Source of Isolation (%) ^#^				Number of Sequences ^*^	Motif ^§^ (Incidence, %)	Geographical Area (%)		
				Chicken	Duck (Domestic)	Wild Duck	Turkey			China	Hong Kong	Taiwan
1–9	MNTQILVFA	92	I (75%)	36	18	11	-	40	I (100%)	98	3	-
	......ALI		Ma (16%)	-	-	-	9		X	-	-	-
	..I......		Mi (2%)	-	-	-	-		X	-	-	-
	......A.I		Mi (2%)	-	-	-	-		X	-	-	-
	........I		Mi (2%)	-	-	-	2		X	-	-	-
	......TLI		U (1%)	-	-	-	-		X	-	-	-
	....V....		U (1%)	-	1	-	-		X	-	-	-
227–235	GARPQVNGQ	99	I (63%)	5	16	12	10	45	X	-	-	-
	........L		Ma (35%)	28	2	-	-		I (93%)	87	2	4
	.T.......		Mi (2%)	-	-	-	-		X	-	-	-
	..G.....L		X	-	-	-	-		U (2%)	2	-	-
	........I		X	-	-	-	-		U (2%)	2	-	-
	....P...L		X	-	-	-	-		U (2%)	2	-	-

† Amino acid number at the start and end of a nonamer position in the protein alignment. HA protein sequence numeration is based on the H7N9 HA protein sequence alignment. Positions 1–9 with identical avian and human index sequences. Positions 227–235 with a major variant of the avian index selected as the human index sequence. ‡ The nonamer sequence of a given position placed at the top is the avian virus index motif; the remaining sequences below are variants of the avian index sequence. Amino acids identical between the index and the variants are indicated with dots. ^ All percentages are shown to the nearest whole number. * Number of sequences analyzed: avian, 101; human, 53; however, the numbers can differ from position to position because of the inclusion of partial sequences, besides full-length, in the alignment. § The index nonamer (I) is the most prevalent sequence at a given aligned nonamer position. The motifs differ by one or more amino acids from the index sequence. The major motif (Ma) is the most common variant sequence at the position. Minor motifs (Mi) are multiple different sequences, each occurring more than once and with an incidence less than or occasionally equal to the major motif. Unique motifs (U) are those that occur only once in the alignment. “X” represents sequences that are absent from the respective host. # Only avian host with at least 10% cumulative incidence of the distinct sequence(s) are shown.

## Data Availability

Public data was used. Information related to the data is provided in the main text and supplementary files.

## Supplementary Materials

The following are available online at https://www.mdpi.com/article/10.3390/v13050871/s1, Figure S1: Hierarchical clustering of avian-to-human substitution for all proteins of influenza A among publicly reported, full-length, avian and human influenza A(H7N9) virus strains, Table S1A: Avian influenza A (H7N9) virus dataset: host species, geographical location and year reported, Table S1B: Location of human influenza A (H7N9) virus protein sequences reported since the year 2013, Table S2: The diversity motifs of avian and human influenza A(H7N9) proteome nonamer positions, Table S3A–D: Avian-to-human (A2H) substitution identified in the proteins of influenza A(H7N9) viruses from 2014 and 2017 datasets, Table S4: Avian-to-human (A2H) substitutions with 100% incidence in human H7N9 viruses and first identified in 2013, Table S5: Amino acid substitutions specific to human influenza A(H7N9) virus, Table S6: Summary of A2H amino acid substitutions’ net motif change between 2014 to 2017 datasets, Table S7: Heat map depicting timeline of adaptation to avian-to-human substitution (A2H) for all proteins of influenza A among publicly reported, full-length, avian and human influenza A(H7N9) virus strains, Table S8: Comparison of the avian-to-human (A2H) substitution identified in the proteins of influenza A(H7N9) viruses from 2014 and 2017 datasets against the internal proteins of H9N2 chicken viruses, reported before and 2013 onward, Table S9: Acknowledgement to the authors, originating and submitting laboratories of the sequences from GISAID’s EpiFlu Database.
