# Supplementary material for: Avian Influenza H7N9 Virus Adaptation to Human Hosts
Source: Viruses. 2021 May 10;13(5):871. doi: 10.3390/v13050871 (PMC8150935; doi:10.3390/v13050871)
Supplement: Supplementary file 1 [file viruses-13-00871-s001.zip › SUPPLEMENTARY FIGURE 1.pdf]

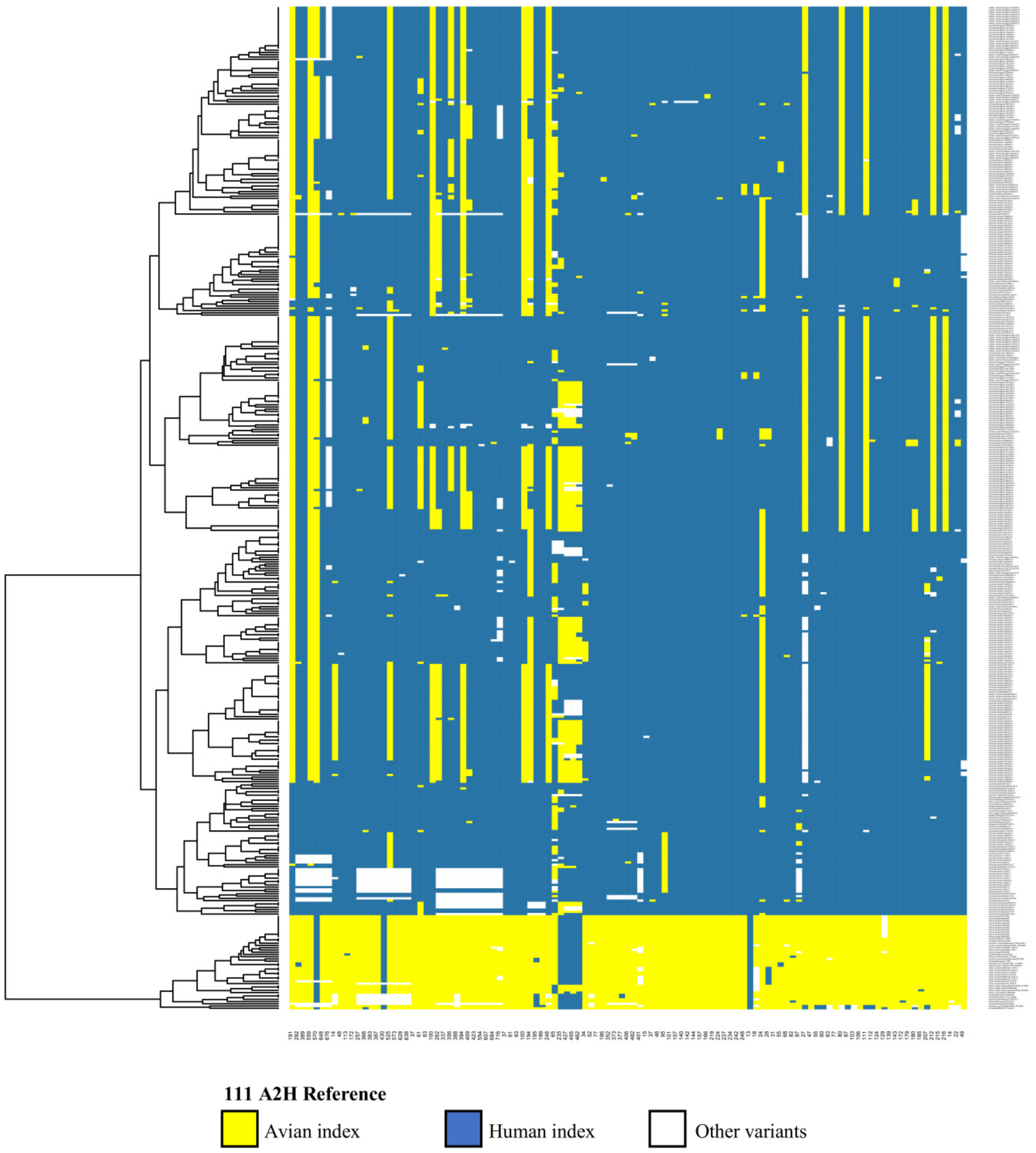

Supplementary Figure 1 | Hierarchical clustering of avian-to-human substitution for all proteins of influenza A among publicly reported, full-length, avian and human influenza A(H7N9) virus strains. The clustering was done by use of the R package "gplots" with default settings. The heatmap shows different species samples in 'row' with a dendrogram representing their similarity based on the 111 amino acid substitution positions. The strains that were isolated before and after 2013 H7N9 outbreak are clearly separated in two big nodes with small multiple branches.
