# Supplementary material for: Avian Influenza H7N9 Virus Adaptation to Human Hosts
Source: Viruses. 2021 May 10;13(5):871. doi: 10.3390/v13050871 (PMC8150935; doi:10.3390/v13050871)
Supplement: Supplementary file 1 [file viruses-13-00871-s001.zip › SUPPLEMENTARY TABLE 1A&B.pdf]

Supplementary Table 1A | Avian influenza A (H7N9) virus dataset: host species, geographical location and year reported.

Host species	Location	Year	Before 2013	After 2013
Anas crecca	Spain	2008	12	-
Blue-winged teal	USA	2006	12	-
	Guatemala	2008	24	-
Chicken	China, Guangdong	2013	-	12
	China, Guangzhou		-	1
	China, Jiangsu		-	50
	China, Jiangxi		-	12
	China, Rizhao		-	56
	China, Shanghai		-	143
	China, Zhejiang		-	51
Duck	China, Anhui	2013	-	12
	China, Gunma	2011	1	-
	China, Jiangxi	2009	132	-
	Mongolia	2008	4	-
		2010	1	-
	China, Zhejiang	2013	-	12
Emperor goose	USA	2006	12	-
Goose	Czech Republic	2009	24	-
	USA	2011	13	-
Guinea fowl	USA	2011	14	-
Homing pigeon	China, Jiangsu	2013	-	12
Mallard	Albania	1999	1	-
	Spain	2005	1	-
	Sweden	2002	13	-
Northern shoverl	USA	2011	12	-
Pigeon	China, Shanghai	2013	-	36
Ruddy turnstone	USA	2000	13	-
		1995	12	-
Spot billed duck	Korea	2011	11	-
Tree sparrow	China, Shanghai	2013	-	23
Turkey	USA	1988	23	-
		2009	69	-
		2011	21	-
Wild bird	Korea	2011	33	-
Wild duck	Korea	2008	12	-
		2010	48	-
		2011	72	-
	Mongolia	2008	9	-
Wild pigeon	China, Jiangsu	2013	-	12
		Subtotal	599	432
		Total	1031	

Supplementary Table 1B | Location of human influenza A (H7N9) virus protein sequences reported since the year 2013.

Location	Since 2013
Changsha	24
Fujian	12
Guangdong	54
Guangzhou	2
Hangzhou	17
Hong Kong	12
Huizhou	12
Jiangsu	36
Nanchang	11
Nanjing	60
Shanghai	71
Suzhou	24
Taiwan	24
Wuxi	48
Xuzhou	12
Zhejiang	48
Zhenjiang	12
**Total**	**479**
