# Supplementary material for: Avian Influenza H7N9 Virus Adaptation to Human Hosts
Source: Viruses. 2021 May 10;13(5):871. doi: 10.3390/v13050871 (PMC8150935; doi:10.3390/v13050871)
Supplement: Supplementary file 1 [file viruses-13-00871-s001.zip › SUPPLEMENTARY TABLE 6.docx]

**Supplementary Table 6 |** Summary of A2H amino acid substitutions' net motif change between 2014 to 2017 datasets.

| Protein | Number of A2H amino acid substitution  (2014 dataset) | Net motif change between 2014 and 2017 datasets^*^ | | |
| --- | --- | --- | --- | --- |
|  |  | Avian | Human | Frequency (count) |
| PB2 | 7 | Reversed | Unchanged | (4) |
|  |  | Unchanged | Reversed | (2) |
|  |  | New | New | (1) |
| PB1 | 13 | Reversed | Unchanged | (12) |
|  |  | Unchanged | Unchanged | (1) |
| PA | 15 | Reversed | Unchanged | (12) |
|  |  | Unchanged | Unchanged | (2) |
|  |  | Reversed | Reversed | (1) |
| PA_X | 8 | Reversed | Unchanged | (3) |
|  |  | Unchanged | Unchanged | (3) |
|  |  | Reversed | Reversed | (2) |
| HA | 5 | Reversed | Unchanged | (4) |
|  |  | New | New | (1) |
| NP | 9 | Reversed | Unchanged | (9) |
| NA | 1 | Reversed | Unchanged | (1) |
| M1 | 17 | Reversed | Unchanged | (17) |
| M2 | 9 | Reversed | Unchanged | (9) |
| NS1 | 24 | Reversed | Unchanged | (21) |
|  |  | Reversed | Reversed | (2) |
|  |  | Unchanged | Unchanged | (1) |
| NS2 | 3 | Reversed | Unchanged | (3) |
| Total | 111 | Reversed | Unchanged | (95) |
|  |  | Unchanged | Unchanged | (7) |
|  |  | Reversed | Reversed | (5) |
|  |  | Unchanged | Reversed | (2) |
|  |  | New | New | (2) |

*The net motif change is shown for each substitution, as per the order in column D. The 'Unchanged' status refers to no motif change in the substitution, whereby the major variant of 2014 remained as a major variant in 2017, in the respective population. The 'New' status refers to an A2H substitution that satisfied the selection criteria (see Methods) in the 2017 dataset, but not the 2014 dataset (shown as N/A). The 'Reversed' status refers to a motif change in the substitution, whereby the major variant is no longer a major variant in the respective population; the change could be due to an increase (index) or decrease (minor or unique) in the motif rank.
